# Quantifying the relationship of HIV infection with clinicopathological spectrum and outcome among patients with colorectal cancer in a South African population

**DOI:** 10.4314/ahs.v22i2.4

**Published:** 2022-06

**Authors:** SK Pillay, Z Moolla, Y Moodley, TE Madiba

**Affiliations:** 1 Gastrointestinal Cancer Research Group, Department of Surgery, University of KwaZulu-Natal, Durban, South Africa; 2 Faculty of Health and Environmental Sciences, Central University of Technology, Bloemfontein, South Africa; 3 African Cancer Institute, Stellenbosch University, Cape Town, South Africa

**Keywords:** Colorectal cancer, colon cancer, rectal cancer, HIV, Outcome

## Abstract

**Introduction:**

Literature is limited on HIV and colorectal cancer (CRC) in sub-Saharan Africa despite it being the epicentre of the HIV epidemic,

**Purpose:**

To compare clinicopathological features and outcome of CRC in HIV-negative and HIV-positive patients.

**Methods:**

Retrospective analysis of a prospective CRC database. Demographic details, HIV status, anatomical site, disease stage, treatment and follow-up were documented.

**Results:**

Of 715 patients with CRC, 145 and 570 tested positive and negative respectively for HIV. Median age was 45 (IQR 36–53 and 57 (IQR 45–66) years among HIV-positive and HIV-negative patients respectively (p<0.0001). Tumour differentiation differed between the two groups (p=0.003) but staging was not different (p=0.6). Surgical resection rate was 52% for HIV-positive patients versus 59% for HIV-negative patients (p=0.07). Median follow-up was 9 (IQR 2–20.5) months for HIV-positive patients and 12 (IQR 6–29) months for HIV-negative patients (p=0.154). Recurrence rate was 14.7% among HIV positive patients and 6.8% in HIV negative patients (p=0.089).

**Conclusion:**

When compared with HIV-negative patients, HIV-positive patients with CRC presented at a younger age and tended to have lower surgical resection rates. There was no difference between the two groups with CRC in terms of anatomical sub-site distribution, disease staging and recurrence rates.

## Introduction

Non-AIDS-defining Cancers (NADCs) are increasingly becoming an important cause of mortality in people with HIV infection 1. While the incidence of AIDS-defining malignancies has decreased, there has been a rise in the incidence of NADCs 1,2, and this increase has continued even after the introduction of Highly Active Antiretroviral Therapy (HAART) 3–7. Colorectal cancer (CRC) is the third most commonly diagnosed malignancy and the fourth leading cause of cancer-related death in the world, accounting for about 1.4 million new cases and almost 700 000 deaths in 2012 8,9.

In South Africa, CRC has moved from being the 10^th^ most diagnosed cancer in the 1990's 10,11, to its current status of being ranked among the top four cancers in both males and females 12,13. The risk of developing colorectal cancer in people living with HIV remains uncertain 3,14 although epidemiological studies have shown no increase in incidence 6,14. South Africa has a dual health system, namely the public health system and the Private health care system. The Public sector comprises government health institutions and serves predominantly indigent population, while the Private sector serves the insured population and those who can afford health care 15. The public sector is responsible for the well-being of 80% of the population, while the private sector is responsible for less than 20% of the population 16,17.

There is sparse literature addressing the clinicopathological spectrum of CRC specifically in patients with HIV infection. Despite sub-Saharan Africa being most affected by the HIV epidemic 18, current literature regarding CRC and HIV has originated from high income countries (HIC) and no studies related to CRC incidence and HIV have been conducted in sub-Saharan Africa. In addition, data from the international literature cannot be directly extrapolated to the African population as the health care situations between HIC and low-and-middle-income countries are vastly different. Furthermore, sample sizes on previous studies on CRC and HIV have been very small, which makes it difficult to reach any conclusions. We hypothesised that there may be an association between the clinicopathological spectrum of colorectal cancer and HIV infection. The aim of this study therefore was to compare the clinicopathological spectrum and subsequent outcome of colorectal cancer in HIV-positive and HIV-negative patients by analysing data extracted from the local ongoing colorectal cancer database. The manuscript was prepared according to the STROBE checklist.

## Methodology

### Study setting

The study was carried out at the Durban Colorectal Unit situated at Inkosi Albert Luthuli Central Hospital (IALCH), a tertiary referral hospital in Durban, South Africa. IALCH serves the Eastern seaboard of the Kwa-Zulu-Natal Province of South Africa, which covers an area of over 92 000 km^2^. It houses the Colorectal and Oncology units, both of which participate in the Gastrointestinal Cancer Multidisciplinary Team (MDT). Additional Colorectal and Oncology units are situated at Addington Hospital (ADH) in Durban and Grey's Hospital (GH) in Pietermaritzburg, both of which are subsidiary to the Main Units at IALCH. All patients with rectal cancer are discussed at the Multidisciplinary Clinic consisting of an MDT of surgeons, oncologists and radiologists. Members of the Colorectal Unit at IALCH are also members of the MDT. The MDT thus collectively decides the proposed treatment plan.

### Colorectal Cancer Database

The ongoing colorectal cancer database commenced in 2000 and is archived in the Gastrointestinal Cancer Research Centre of the University of KwaZulu-Natal. New patients were identified at the initial presentation at the three hospitals with Colorectal and Oncology services and/or at the time of arrival at the various Oncology Departments. Follow-up data are collected from the Colorectal and Oncology records and entered onto the database.

### Study population

All patients with colorectal cancer referred to IALCH are entered onto a colorectal cancer database. Data of patients diagnosed with rectal cancer between 2000 and 2019 were extracted from the database and analysed. Patients with disease at multiple sites were excluded from this analysis. The enrolment into the database is dependent on the patents' presentation at the UKZN-affiliated hospitals. Patients with colonic cancers are generally managed surgically at the regional hospitals and are referred to IALCH, where they are seen by the Multidisciplinary Team (MDT), only after resection; the exception is for patients with complicated disease that requires management in a central hospital. Patients with rectal cancers are referred to the MDT before treatment for management decision by the team. Patients who present with acute complete colonic obstruction undergo emergency laparotomy at the referring hospital where a defunctioning colostomy is performed. If the obstruction is partial, the patient is referred to the Colorectal Unit where they are then assessed for eligibility of either stent insertion or diverting colostomy. Population groups are defined as African, Indian, Coloured, and White according to the criteria used by the South African Government 19,20. In South Africa, “Coloured” refers to people of mixed ancestry 20.

### Study design

This was a retrospective analysis of prospectively collected data. Convenience sampling was employed involving patients with colorectal cancer from 2000 to 2019 extracted from the Colorectal Cancer Database. Voluntary Counselling and Testing (VCT) was not routinely offered in the early years of the database due to the stigma associated with HIV infection and there was consequently sporadic recording of HIV status. Some patients had HIV tests done prior to admission at IALCH clinic, so the HIV status was known at that point. In those patients who did not have HIV status known, VCT was offered and, when accepted, VCT was performed by dedicated personnel at IALCH; thereafter the HIV sero-status was determined by drawing a vial of patients' blood and sending this to an accredited virological laboratory adjoining the hospital for testing. Data of patients with a known HIV status were extracted from the database and analysed for comparison between HIV positive and negative patients. Proximal colon was defined as the colonic segment from the caecum up to and including the transverse colon and the distal colon was the segment extending from the splenic flexure to and including the sigmoid colon 21. The surgeons in our unit adopted and employed the principles of total mesorectal excision 22. The surgical procedure was performed via the open or laparoscopic approach. The TNM and UICC staging systems were used. The T and N stage was based on examination of the resected specimen. The M stage could be assessed with or without resection by virtue of the presence of metastasis. The UICC staging is reported in this paper.

### Data management and analysis

The following information was collected onto the database, namely demographics, clinical presentation, staging, treatment and follow-up. At IALCH all the patient details which include demographic details, stage of the disease, site of disease, years of survival, months of follow-up, and treatment are captured. The data were captured onto Microsoft Excel® and data analysis was conducted using the Statistical Package for the Social Sciences (SPSS) version 26.0 (IBM Corp., USA). The data was tested for normality (Gaussian distribution) but failed the Shapiro-Wilk test (p<0.05) and hence the results were analysed and presented as medians with IQR. Subgroup analysis for age was analysed among African patients only using the Wilcoxon test. For the comparison of proportions (%), the Chi-squared test was employed. To allow for comparisons between groups, post-hoc testing was applied. A p value of <0.05 was considered statistically significant.

### Ethical considerations

Informed consent was obtained prior to patient enrollment. Other clinical data were collected from the clinical records. Confidentiality was maintained by de-identifying patients in the database. Ethical approval for the study was granted by the University of KwaZulu-Natal Biomedical Research and Ethics Committee (Ref No.: E198/04).

## Results

At the time of analysis, the database contained the entries of 2523 patients with CRC cancer. The demographics of all the patients with colorectal cancer are shown in Table I. African patients had the youngest median age at presentation and had the highest proportion of patients of age < 40 years. Seven hundred and fifteen patients (28.3%) either underwent voluntary counselling and testing (VCT) or had an already established diagnosis of HIV. Five hundred and seventy participants (79.7%) tested HIV negative whilst 145 tested HIV positive (20.3%). The proportion of patients who tested positive for HIV was highest among African patients at 30.4% and that for the other population groups ranged between three and eight percent. The median CD4 count was 400 (IQR 212–500) cells/mm^3^.

**Table I:** Profile Of Patients With Colorectal Cancer Stratified According To Age And HIV Status

Parameter	Total with CRC	Age in years (IQR)	Age ≤ 40 years n (%)	Total undergoing VCT	HIV positive n (%)
Total	2523	59 (49–68 )	334 (12.2%)	715	145 (20.3%)
African	948	52 (40–63 )	248 (26.2%)	434	132 (30.4%)
Indian	986	61 (53–68)	61 (6.2%)	175	5 (2.9%)
Coloured	116	60 (53–68.5)	10 (8.6%)	33	2 (6%)
White	473	67 (58–74)	15 (3.2%)	73	6 (8.2%)

VCT = Voluntary counselling and testing, IQR = Interquartile range

Table II shows the profile of HIV positive and negative patients with CRC. The HIV positive population presented at a median age of 45 (IQR 36–53) years of age, whereas the HIV negative population presented a decade later, at a median age of 57 (IQR 45–66) years (p<0.0001).

**Table II:** Demographic Profile Of HIV Positive And Negative Patients With Colorectal Cancer

Parameter	HIV Positive	HIV Negative	P
Total	145	570	
Median age in years (IQR)	45 (36–53)	57 (45–66)	<0.0001
≤ 40 years	56 (38.6%)	100 (17.5%)	<0.0001
Male	55 (37.9%)	320 (56%)	<0.001
M:F ratio	1:1.6	1.3:1	<0.001
Tumour complications	44 (31%)	151 (26.7%)	0.334
Resection	75 (51.7%)	338 (59.3%)	0.07

IQR = Interquartile range

**Figure 1:**
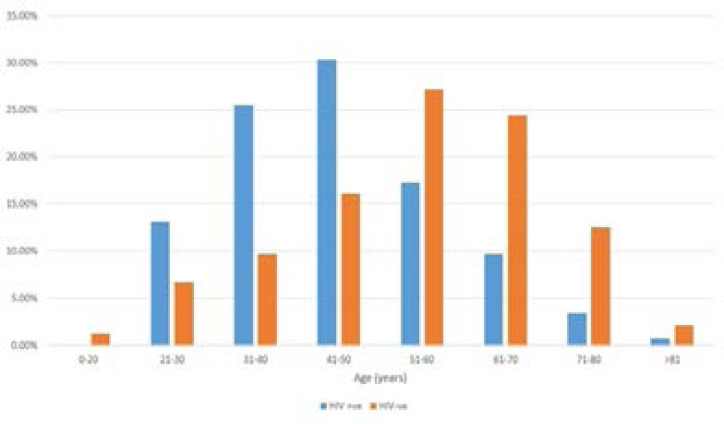
Age distribution of HIV positive and negative patients with colorectal cancer

As shown in Figure 1, the peak age for HIV-positive patients was in the fifth decade, whereas that for HIV-negative patients was in the sixth decade. Female patients predominated among HIV positive patients, whereas males predominated among HIV negative patients (p<0.001).

Table III shows a subgroup analysis for age but the statistical analysis was performed only for African patients. When the two groups were statistically compared the HIV positive group was significantly younger than the HIV negative group (p<0.001). Subgroup analysis among the other population groups was not done because of very small numbers.

**Table III:** HIV Status Stratified According To Populatio n Groups

Population Group	HIV positive n= 145		HIV negative n= 570		P value
	n (%)	Age [n(IQR)]	n (%)	Age [n(IQR)]	p
African(n=427)	132 (30.9%)	44 (34–51)	298 (69.8%)	52 (39–63)	<0.001
Indian (n=176)	5 (3.4%)	68 (58–69)	170 (96.6%)	59.5 (52–67)	-
Coloured (n=33)	2 (6%)	54.5 (42–67)	31 (93.9%)	57 (52–62)	-
White (n=72)	6 (8.3%)	68 (58–74)	66 (91.7%)	65 (57–71)	-

* The subgroup analysis for age was performed among African patients only.

IQR = interquartile range

As can be seen in Table IV there was no difference in sub-site distribution of colorectal cancer in HIV-positive and HIV-negative patients. However, there was a trend towards predominance of rectal disease among HIV positive patients (p=0.344). Table V shows tumour staging and tumour differentiation in the study sample. There was a trend for a higher proportion of patients with Stage IV disease among HIV-positive patients and that of stage III in HIV-negative patients. Despite these trends, there was no statistically significant difference in staging between the two groups. There was a statistically significant difference between the HIV positive and negative patients in terms of tumour differentiation (p=0.003). A higher proportion of poorly differentiated carcinoma was noted among HIV-positive patients compared to HIV negative patients. The other grades of differentiation were similar in both groups.

**Table IV:** Site Distribution Of Colorectal Malignancy In Relation To HIV Status

Site	HIV Positive n=145	HIV Negative n=570	p
***Proximal vs distal colon***			
Proximal colon	33 (22.9%)	121 (21.2%)	0.288
Distal colon	24 (16.6%)	140 (24.6%)	
***Colon vs rectum***			
Colon	57 (39.3%)	261 (45.8%)	0.344
Rectum	87 (60%)	301 (52.8%)	

Patients with involvement of multiple sites were excluded from this analysis

**Table V:** Staging And Differentiation In HIV Positive And Negative Patients With Colorectal Cancer

**Staging**			
**Stage**	**HIV Positive** **(n=145)**	**HIV Negative** **(n=570)**	**p**
I	9 (6.2%)	37 (6.4%)	0.6
II	17 (11.7%)	106 (18.6%)	
III	29 (20%)	145 (25.4%)	
IV	46 (31.7%)	152 (26.7%)	
Unknown	44 (30.3%)	130 (22.8%)	
**Differentiation**			
	**HIV Positive** **(n=145)**	**HIV Negative** **(n=570)**	**p**
Moderate	81 (55.9%)	390 (68.4%)	0.003
Mucinous	9 (6.2%)	41 (7.2%)	
Poor	15 (10.3%)	21 (3.7%)	
Well	1 (0.7%)	9 (1.6%)	
Undifferentiated	0	1 (0.2%)	
Not stated	38 (26.2%)	107 (18.8%)	

**Figure 2:**
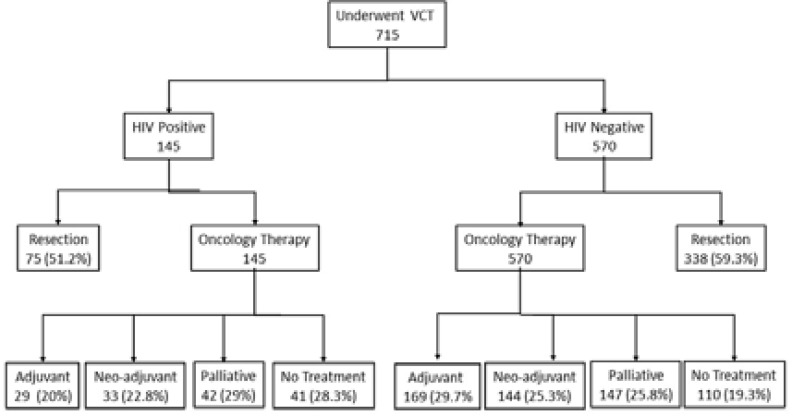
Flow diagram showing sample derivation patients with colorectal cancer stratified according to HIV status

Treatment of CRC is shown in the flow diagram in Figure 2. Twenty-eight percent and 19% respectively did not receive oncological therapy in both groups for various reasons including failure to present, poor general condition, and defaulting treatment. Seventy-five patients among the HIV positive group (51%) underwent resection compared to 338 (59%) HIV negative patients (p =0.07). Twenty-two HIV positive patients (15.2%) and 81 HIV negative patients (14.2%) were lost to follow-up soon after diagnosis. Among the patients that were available for follow-up, there was a high attrition rate but the median follow-up ended up being 9 (IQR 2–20.5) months for HIV positive patients and 12 (IQR 6–29) months (p=0.154). Recurrent disease occurred in 11 of 75 patients (14.7%) among HIV positive patients and in 23 of 338 patients (6.8%) in HIV negative patients (p=0.089). disease-free interval was 25 months (IQR 36–53) for HIV positive patients and 15 months (IQR 9–28). Survival rate among the 10 patients whose death dates were known was 19.5 months (IQR 12–30) among HIV positive patients and 11 months (IQR 4–25) among 35 HIV negative patients.

## Discussion

This study has several key findings. When considering the overall cohort, African patients tended to be younger than the other population groups; an observation which has been made by other South African authors^23^,^24^. Among the study cohort, HIV positive patients tended to present at a younger age compared to their HIV negative counterparts. Subgroup analysis demonstrated that, even among African patients (who are generally younger overall), HIV positive patients were younger than HIV negative patients. International studies have similarly shown that HIV infected individuals present at a younger age in most cancers such as lung, liver, anal and breast^2^,^25^ and more specifically in CRC 26–28. It is tempting to speculate that this presentation at a young age may be related to accelerated aging seen in HIV-infected individuals 29,30, or gut flora dynamics in the HIV-positive patient 30–33. We concede, however, that a possible major confounder is the fact that colorectal cancer tends to present at a younger age in black Africans 23,24, who also have a higher frequency of HIV infection in South Africa 34. Thus, it is possible that the younger age at presentation of HIV infected patients with colorectal cancer is simply because there were more black Africans in this group.

The gender profile of HIV positive and negative patients with CRC differed between the two groups with females predominating in HIV-positive patients whereas HIV-negative patients demonstrated a male preponderance. This contradicts findings in international studies which report male predominance in HIV positive patients with CRC 7,27,35. This female preponderance is not surprising in the South African context since various surveys show that women continue to account for a disproportionate percentage of new HIV infections among adults in sub-Saharan Africa (predominantly through heterosexual transmission) where they comprise up to 59% of the affected population 18,36. This gender disparity is possibly related to the lower likelihood of men than women to use health services and the fact that men are less likely to take an HIV test 36,37. Furthermore, there is evidence to suggest that women are susceptible to HIV and other sexually transmitted diseases because of because of a complex set of interrelated biological differences in susceptibility, reduced sexual autonomy, and men's sexual power and privilege 37,38. The higher prevalence of HIV in males with CRC in HIC is more likely related to the predominant modes of HIV transmission in those countries, which are intravenous drug use and men who have sex with men 18. International studies have drawn conflicting results with regard to anatomical distribution of CRC in HIV positive and negative patients, with some authors showing a high incidence of right-sided tumours in HIV positive patients^5^,^6^,^28^, whereas others show predominance of left-sided disease 6,26. The present study showed no particular trends in sub-site distribution in both patient groups.

There are conflicting data on the level of advancement of CRC among HIV positive and negative patients. Some authors contend that HIV-infected individuals present with advanced disease and aggressive course of illness^3^–^5^,^7^,^14^,^28^, while others show similar staging in both HIV positive and negative patients 6,7,26. There was a trend towards a high proportion of stage IV disease in the HIV positive group in this series but the difference was not statistically significant. Interestingly, Sigel et al found no difference in tumour grade or stage by HIV status 6. It is tempting to postulate that the higher proportion of poorly differentiated carcinoma in HIV positive patients may be indicative of a likely aggressive biology in HIV positive patients. We are, nonetheless, cognisance of the fact that these parameters are not the only ones that would predict tumour behaviour.

The surgical resection rate for CRC in HIV positive patients was lower than that in HIV negative patients (53% vs 60%). This may suggest the HIV positive patients may tend to present at an advanced stage of the disease, thus reducing the likelihood of eligibility for resection. There is a need to investigate if this presentation with advanced disease is related to disease aggressiveness, as no published studies have addressed the resection in HIV positive and negative patients.

There was a high attrition rate in both groups being followed up for CRC but it was more pronounced among HIV positive patients. There is evidence to suggest that survival is much poorer in HIV-positive patients when compared with the general population, even following the introduction of HAART 1,4,6,26. Studies show that HIV-infected patients with cancer experienced higher cancer-specific mortality than HIV-uninfected patients, independent of cancer stage or receipt of cancer treatment 39. This high mortality is partly owing to death as a result of AIDS-related complications, but additional proposed explanations for the disparity in survival rates include advanced stage at cancer diagnosis, biologically aggressive cancer phenotype because of immune dysregulation, and decreased efficacy or increased toxicity from cancer therapy 1. These studies suggest that the difference is not only related to advanced tumour stage or lack of cancer treatment but also reflects an effect of immunosuppression on cancer outcomes 39. Because of the high attrition rate in this series, we were not able to perform survival curves. The observations in this study underscore the need for HIV-infected patients with colorectal cancer to be more closely monitored during follow-up.

This study does have some limitations. The CRC database reflects a single academic institution and the affiliated hospitals and thus cannot be generalised to the rest of South Africa. There are some CRC patients who attended private healthcare facilities and these patients are not included in this study. Therefore, while this study might be representative of the majority of CRC patients in the province of KwaZulu-Natal, it does not reflect all CRC patients in the province. HIV status was not routinely checked in the early years of the Colorectal Unit and HIV status was not known in a large number of patients. There was a high attrition rate in both groups, and this is a potential source of bias in the current study. Data on the use of HAART as well as chemotherapy and radiotherapy tolerance were not always captured in this study and this is the basis of an ongoing study in our unit. Although we have referred to VCT in this paper, we concede that the concept of VCT is now out-dated in HIV care and that current recommendations suggest the approach of provider initiated opt-out strategies to testing (PITC) 40.

In conclusion, HIV positive patients with CRC presented at a younger age compared to HIV negative patients. There was a female predominance in the HIV positive population with CRC. No difference in colorectal cancer staging was observed between HIV-positive and HIV-negative groups. A trend towards a lower surgical resection rate for CRC was observed in HIV positive patents when compared with HIV-negative patients with CRC.
